# Partial Depletion of Gamma-Actin Suppresses Microtubule Dynamics

**DOI:** 10.1002/cm.21096

**Published:** 2013-01-17

**Authors:** Sela T Po'uha, Stephane Honore, Diane Braguer, Maria Kavallaris

**Affiliations:** 1 Children's Cancer Institute Australia, Lowy Cancer Research CentreUNSW, Randwick, NSW, Australia; 2 INSERM UMR 911, Centre de Recherche en Oncologie Biologique et en OncopharmacologieAxi-Marseille Université, 27 Boulevard Jean Moulin, 13385 Marseille Cedex 5, France; 3 Australian Centre for Nanomedicine, University of New South WalesSydney, Australia

**Keywords:** γ-actin, microtubule dynamic instability, paclitaxel, neuroblastoma cells, mitosis

## Abstract

Actin and microtubule interactions are important for many cellular events, however these interactions are poorly described. Alterations in γ-actin are associated with diseases such as hearing loss and cancer. Functional investigations demonstrated that partial depletion of γ-actin affects cell polarity and induces resistance to microtubule-targeted agents. To determine whether γ-actin alterations directly affect microtubule dynamics, microtubule dynamic instability was analyzed in living cells following partial siRNA depletion of γ-actin. Partial depletion of γ-actin suppresses interphase microtubule dynamics by 17.5% due to a decrease in microtubule shortening rates and an increase in microtubule attenuation. γ-Actin partial depletion also increased distance-based microtubule catastrophe and rescue frequencies. In addition, knockdown of γ-actin delayed mitotic progression, partially blocking metaphase–anaphase transition and inhibiting cell proliferation. Interestingly, in the presence of paclitaxel, interphase microtubule dynamics were further suppressed by 24.4% in the γ-actin knockdown cells, which is comparable to 28.8% suppression observed in the control siRNA treated cells. Paclitaxel blocked metaphase–anaphase transition in both the γ-actin knockdown cells and the control siRNA cells. However, the extent of mitotic arrest was much higher in the control cells (28.4%), compared to the γ-actin depleted cells (8.5%). Therefore, suppression of microtubule dynamics by partial depletion of γ-actin is associated with marked delays in metaphase-anaphase transition and not mitotic arrest. This is the first demonstration that γ-actin can modulate microtubule dynamics by reducing the microtubule shortening rate, promoting paused/attenuated microtubules, and increasing transition frequencies suggesting a mechanistic link between γ-actin and microtubules. © 2013 Wiley Periodicals, Inc

## Introduction

The microfilaments and microtubules are a major component of the cytoskeletal network and play crucial roles in cell division, cell motility, vesicular transport, and maintenance of cell shape. Microtubules are highly dynamic at their plus ends, which alternate between phases of growing, rapid shortening, and a phase of attenuated dynamics or pause. This unique behavior of microtubules called “dynamic instability” is useful for the rapid organization and reorganization of the cytoskeleton during cell division and cell migration. Microtubule dynamic instability is characterized by at least six parameters which are the growing and shortening rates, the catastrophe and rescue frequencies, the mean duration of pause and the overall dynamicity which represent the overall exchange of tubulin at the plus end of the microtubule per unit time. Microtubule dynamic instability changes throughout the cell cycle; microtubules are less dynamic during interphase and highly dynamic during mitosis [Kavallaris, 2010]. Regulation of microtubule dynamics is important in mitotic spindle assembly and function [Saxton et al., 1984]. Several proteins can modulate microtubule dynamic instability by either stabilizing, or destabilizing, the microtubule including the microtubule-associated proteins (MAPs) such as MAP2 [Itoh and Hotani, 1994; Itoh et al., 1997], MAP4 [Ookata et al., 1995], Tau [Drechsel et al., 1992], and stathmin [van der Vaart et al., 2009] and the microtubule plus-end tracking proteins (+TIPS) such as EB1, CLIP170, and XMAP215 [Carvalho et al., 2003; Howard and Hyman, 2003; van der Vaart et al., 2009].

The cross-talk between actin filaments and microtubules is crucial for cell division and cell migration [Rodriguez et al., 2003], and dynamic interactions between these two cytoskeletal networks is tightly regulated. It is unclear whether this interaction between the actin filaments and microtubules is of importance for regulation of microtubule dynamics. The non-muscle actin consists of two main isoforms, β- and γ-actin which differ by four amino acids at the N-terminus. However from previous published data, β- and γ-actin have been shown to have distinct localization and unique function [Hill and Gunning, 1993; Dugina et al., 2009; Baranwal et al., 2012]. We recently showed that in neuroblastoma cells the non-muscle γ-actin is organized to a fine actin meshwork and localized to the cell periphery, near lamella and lamellipodial-like structure, whereas β-actin is mainly localized to the stress fibers [Shum et al., 2011]. Generation of knockout mice revealed that β-actin is essential for embryonic development since β-actin knockout (Actb^−/−^) mice were non-viable [Bunnell et al., 2011; Tondeleir et al., 2012]. On the other hand, γ-actin is not required for development since γ-actin knockout (Actg^−/−^) mice were viable, however these mice have reduced viability and suffer hearing loss in adulthood [Belyantseva et al., 2009]. Importantly, alterations in γ-actin have been implicated in human diseases such as hearing loss [Zhu et al., 2003; Bryan et al., 2006], Baraitser-Winter syndrome [Riviere et al., 2012] and drug resistance in cancer [Verrills et al., 2003, 2006a, b].

Recent findings have suggested a link between γ-actin and microtubules. Impaired cell migration, motility, and polarity due to suppression of γ-actin expression was first reported in fibroblast cells [Dugina et al., 2009]. We recently demonstrated in cancer cells that partial depletion of non-muscle γ-actin reduced neuroblastoma cell migration and motility and induced defects in cell polarity [Shum et al., 2011]. Moreover, alterations in the expression of non-muscle γ-actin and actin-associated proteins are associated with resistance to microtubule-targeted agents in childhood leukemia cells [Verrills et al., 2003, 2006b]. Functional studies showed that partial depletion of non-muscle γ-actin in neuroblastoma cells conferred resistance specifically to microtubule-targeted agents and protected the microtubules from disruption by microtubule-targeted agents [Verrills et al., 2006b]. Based on these studies we hypothesized that altered γ-actin expression perturbs microtubule dynamics thereby influencing the action of microtubule-targeted agents. To address this hypothesis, functional and structural studies using gene silencing and time-lapse fluorescence microscopy were performed.

## Results

### Partial Depletion of γ-Actin Perturb Microtubule Structure

**Fig. 1:**
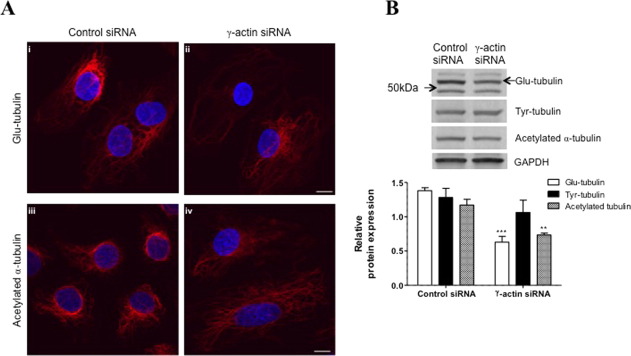
Assessing microtubule structure in the γ-actin knockdown cells (**A**) The SH-EP GFP-βI-tubulin cells were transfected with either the control siRNA or γ-actin siRNA for 72 h then immunostained with detyrosinated (Glu-tubulin) (i and ii) and acetylated-tubulin (iii and iv) antibodies. Images were taken using a laser scanning confocal microscope. Scale bar, 10 μm. (**B**) Western blotting analysis for the expression of Glu-tubulin, tyrosinated (Tyr) and acetylated tubulin in the siRNA transfected cells. GAPDH was included as a protein control for equal loading. Graph showing quantitative analysis of protein expression. Data are mean ± SEM of at least four independent experiments. ^**^P < 0.005, ^***^P < 0.001 statistically significant comparing γ-actin knockdown cells to the control siRNA cells.

Initially we determined whether γ-actin knockdown induced changes in microtubule structures. Immunostaining and immunoblotting were carried out with acetylated, tyrosinated (Tyr-tubulin) and detyrosinated (Glu-tubulin) α-tubulin antibodies to study the relative stability of the microtubules in the γ-actin knockdown cells. Immunostaining with Glu-tubulin detected only a subset of the microtubules originating from the microtubule organizing center (MTOC) in a sinuous appearance but did not extend to the cell periphery in either the control siRNA or γ-actin knockdown cells (Fig. 1Ai and ii). This type of appearance has been previously described in African green monkey kidney cells, TC-7 [Gundersen et al., 1984]. Pronounced Glu-tubulin staining was observed around the nucleus of the control siRNA cells (Fig. 1Ai) whereas some of the γ-actin knockdown cells displayed long Glu-microtubules radiating from the MTOC, and some cells have few microtubules that are detyrosinated (Fig. 1Aii). Similar results were observed when staining the microtubules with acetylated tubulin antibody (Fig. 1Aiii and iv). There was no difference in the Tyr-microtubules between the control siRNA and the γ-actin knockdown cells (data not shown). Expression of Glu-tubulin and acetylated tubulin were decreased in the γ-actin knockdown cells by 54% and 38%, respectively (Fig. 1B), with no significant alteration in Tyr-tubulin levels compared to the control siRNA cells (Fig. 1B). These observations were also confirmed with two independent siRNAs, γ-actin siRNA duplex 1 and 2 that suppressed γ-actin expression by 54% and 63%, respectively, compared to the control siRNA transfected cells (Supporting Information Fig. S1). Immunostained images clearly showed less detyrosinated and acetylated microtubules in the γ-actin knockdown cells compared to the control siRNA cells (Supporting Information Fig. S2A). Western blot analysis revealed a significant decrease in detyrosinated (Glu) tubulin by 48% and 45% for γ-actin siRNA duplex 1 and 2, respectively and a trend toward decreased acetylated tubulin in the γ-actin knockdown cells (Supporting Information Fig. S2B). Knockdown of γ-actin altered microtubule structures and was associated with decreased levels of microtubule acetylation and detyrosination in SH-EP GFP-βI-tubulin cells. Modification of α-tubulin either by acetylation or detyrosination is associated with microtubule stability. More stable microtubules become increasing detyrosinated [Gundersen et al., 1987]. Microtubules that are highly detyrosinated are less dynamic and are resistant to nocodazole depolymerization [Kreis, 1987], and microtubules that are highly acetylated are resistant to depolymerization [Piperno et al., 1987; Jaulin and Kreitzer, 2010]. Our data indicate an association between γ-actin and microtubule stability and perhaps, microtubule dynamics.

### Knockdown of γ-Actin Suppresses Interphase Microtubule Dynamics

In the preceding section partial depletion of γ-actin altered microtubule structure, therefore to address the possibility that loss of γ-actin expression may perturb the action of microtubule-targeted agents by modulating microtubule dynamics we examined the role of γ-actin in this process. Initially, western blotting was performed to assess the efficacy of the fluorescent labeled γ-actin siRNA in suppressing γ-actin expression in SH-EP GFP-βI-tubulin cells. The level of knockdown was ∼40% (Supporting Information Fig. S3A), with ∼100% transfection efficiency (Supporting Information Fig. S3B) which is consistent with our previous findings using non-fluorescent tagged siRNA in SH-EP cells [Verrills et al., 2006b].

**Fig. 2:**
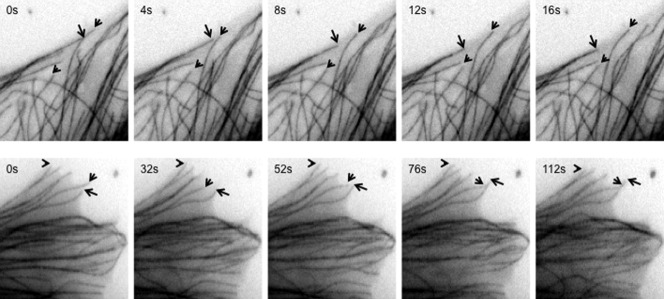
Time-lapse sequences of plus ends of several microtubules in the control (top panels) and the γ-actin knockdown cells (bottom panels) The arrowheads in the control indicate microtubules that grew and shorten over a period of 16 s. The arrowheads in the γ-actin knockdown cells indicate microtubules that shorten and have long paused then underwent a growing event or microtubules that remain unchanged over a period of 112 s.

To examine the effect of γ-actin knockdown on interphase microtubule dynamics, SH-EP GFP-βI-tubulin cells were transfected with either the rhodamine-labeled control siRNA or the γ-actin siRNA for 72 h. The cells were prepared in double-sided coverslip chambers containing recording media and time-lapse images were acquired using a Leica DM-IRBE fluorescence microscope as outlined in “Materials and Methods” section. The plus ends of individual microtubules were followed over time using the Metamorph software as shown in Fig. 2. The plus ends of microtubules (marked by arrowheads) in the control siRNA cells (Fig. 2, top panels) alternate between phases of growing, shortening and paused more frequently compared to the γ-actin knockdown cells where the plus ends of their microtubules were unaltered over a prolonged time (Fig. 2, bottom panels). Partial depletion of γ-actin decreased the microtubule shortening rate (10.65 ± 0.29 μm/min; −15.10%) compared to microtubules in the control siRNA cells (12.55 ± 0.50 μm/min) (Table I). Although the microtubules in the control and γ-actin knockdown cells grew at a similar rate, 8.42 ± 0.23 and 8.45 ± 0.43 μm/min, respectively, the average growing length was shorter in the γ-actin knockdown cells (0.54 ± 0.04 μm; −20.91%) compared to the control siRNA cells (0.68 ± 0.03 μm) (Table I). In addition, knockdown of γ-actin decreased time spent in growing events by 20.11%, which leads to increased time spent in attenuation/pause by 20.39% (Table I). Furthermore, knockdown of γ-actin caused the plus ends of the microtubules to have longer pause duration (+20.26%) compared to microtubules in the control siRNA cells (Table I). Time-based catastrophe and rescue frequencies were unaltered in the γ-actin microtubules compared to the control siRNA cells. In contrast, knockdown of γ-actin increased the distance-based catastrophe and rescue frequencies by 30.86% and 25.21%, respectively. Collectively, alterations in these parameters resulted in suppression of the interphase microtubule dynamicity by 17.45% (4.99 ± 0.22 μm/min, *P* < 0.005) in the γ-actin knockdown cells compared to the control cells (6.05 ± 0.18 μm/min) (Table I). Knockdown of γ-actin decreased interphase microtubule dynamicity suggesting a mechanistic link between γ-actin and microtubule dynamics. Since microtubule dynamics is important for regulating mitosis, suppression of microtubule dynamic instability observed in the γ-actin knockdown cells may perturb mitotic progression.

**Table I:** Changes in Several Parameters of Microtubule Dynamics Induced by Partial Depletion of γ-Actin

Parameter	siRNA transfected cells		
	
	Control	γ-Actin	% Change^a^
Mean rates (μm/min)			
Growth	8.42 ± 0.23	8.45 ± 0.34	NS
Shortening	12.55 ± 0.50	10.65 ± 0.29**	**−15.10**
% Time spent			
Growth	38.40 ± 0.73	30.68 ± 0.90***	**−20.11**
Shortening	22.73 ± 1.27	22.53 ± 0.86	NS
Attenuation	38.87 ± 1.6	46.79 ± 1.39***	**+20.39**
Mean duration (min)			
Attenuation	0.17 ± 0.01	0.20 ± 0.01**	**+20.26**
Mean length (μm)			
Growth	0.68 ± 0.03	0.54 ± 0.04*	**−20.91**
Shortening	0.63 ± 0.05	0.52 ± 0.04	NS
Catastrophe frequency (per min)	2.21 ± 0.17	2.27 ± 0.15	NS
Rescue frequency (per min)	6.84 ± 0.29	7.26 ± 0.23	NS
Catastrophe frequency (per μm)	0.59 ± 0.04	0.78 ± 0.04**	**+30.86**
Rescue frequency (per μm)	0.60 ± 0.05	0.75 ± 0.03*	**+25.21**
Dynamicity (μm/min)	6.05 ± 0.18	4.99 ± 0.22**	**−17.45**

Values are mean ± SEM.

* *P* < 0.05,

** *P* < 0.005,

*** *P* < 0.001, statistically significant when comparing the γ-actin to the control siRNA transfected cells; NS: not statistically significant. Number of microtubules measured was 360 from 90 cells in each condition.

a Percentage change was calculated relative to the control siRNA transfected cells.

### Reduced γ-Actin Expression Delays Mitotic Progression

**Fig. 3:**
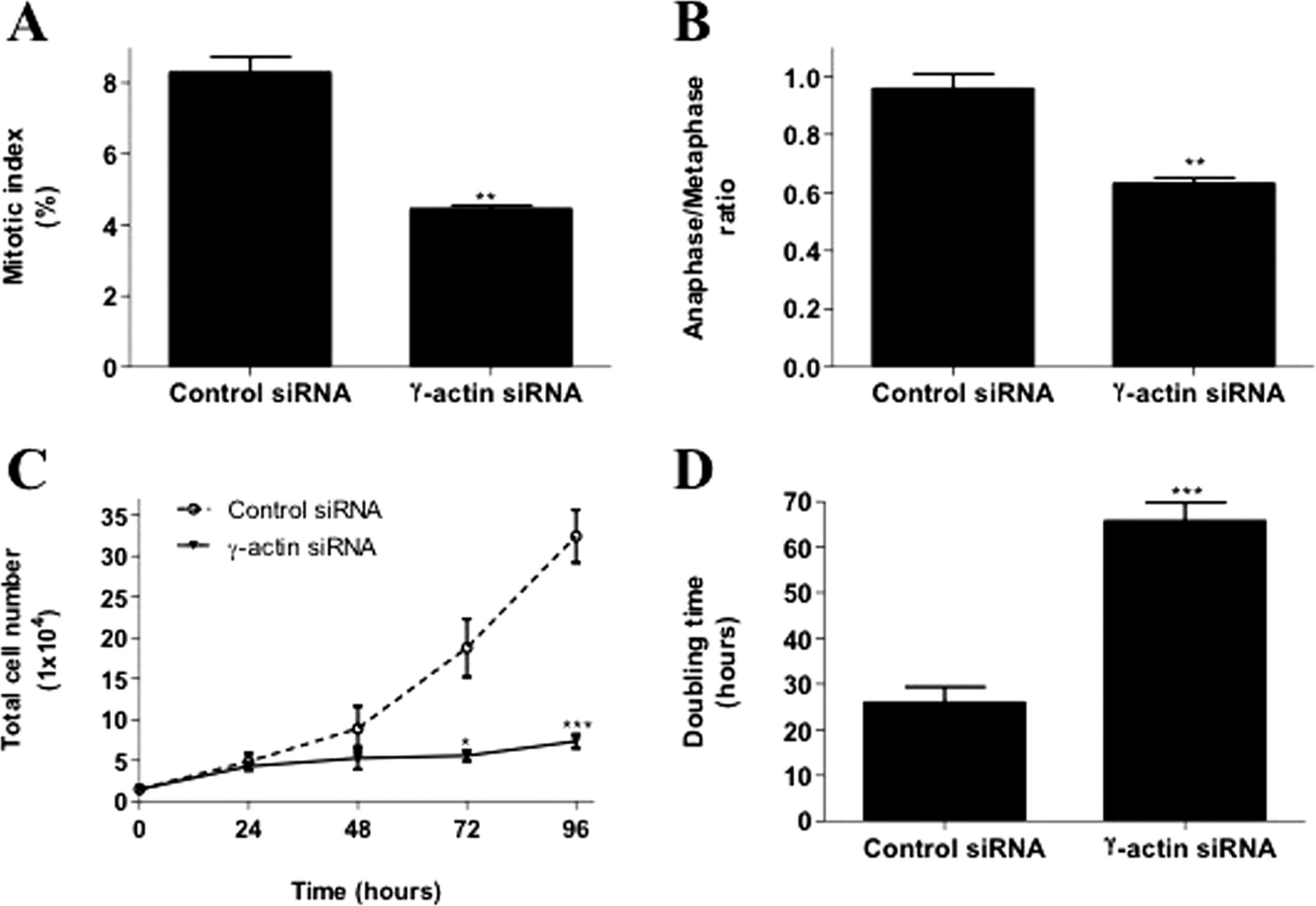
Effect of γ-actin knockdown on mitotic progression and cell proliferation The SH-EP GFP-βI-tubulin cells were either transfected with control siRNA or γ-actin siRNA. Following 72 h post-siRNA transfection, the cells were fixed and stained with DAPI to visualize the DNA. At least 1000 interphase and mitotic cells were counted per condition. (**A**) Mitotic index in the γ-actin knockdown and control siRNA cells. Results are expressed as mitotic index, which was calculated by dividing the total number of mitotic cells by the total number of cells counted. (**B**) Anaphase/metaphase ratio in the γ-actin knockdown and control siRNA cells. (**C**) Growth curves for the control and γ-actin knockdown cells. (**D**) Doubling times for the control and γ-actin siRNA transfected cells. Data are mean ± SEM of at least three independent experiments. *P < 0.05, ^**^P < 0.005, ^***^P < 0.001, statistically significant comparing γ-actin knockdown cells to the control siRNA cells.

It has been previously shown that mitotic arrest induced by microtubule targeted agents is a consequence of suppression of microtubule dynamics [Jordan and Wilson, 2004]. To determine whether suppression of microtubule dynamics in the γ-actin knockdown cells leads to mitotic arrest, mitotic progression was examined in the absence or presence of a microtubule inhibitor. Partial depletion of γ-actin significantly reduced the mitotic index by 1.8-fold (4.45 ± 0.09%, *P* < 0.005) compared to the control siRNA cells (8.29 ± 0.44%) (Fig. 3A). This decrease in mitotic index was accompanied by a delay in metaphase–anaphase transition, as shown by the lower anaphase/metaphase ratio in the γ-actin knockdown cells (ratio 0.63 ± 0.02, *P* < 0.005) compared to the control siRNA cells (ratio 0.96 ± 0.05) (Fig. 3B), a 1.5-fold decrease. This delay in metaphase–anaphase transition is possibly due to knockdown of γ-actin impeding cell proliferation (Fig. 3C), which is reflected by the longer doubling time in the γ-actin knockdown cells (65.6 ± 4.1 h) compared to the control siRNA cells (25.9 ± 3.4 h) (Fig. 3D). This is consistent with previous data on mouse embryonic fibroblasts obtained from γ-actin knockout (Actg^−/−^) mice shown to have impaired cell growth and survival [Bunnell and Ervasti, 2010]. Collectively, these data show that decreased γ-actin expression delays transition from metaphase to anaphase which is reflected by delay in mitotic progression and cell proliferation.

**Fig. 4:**
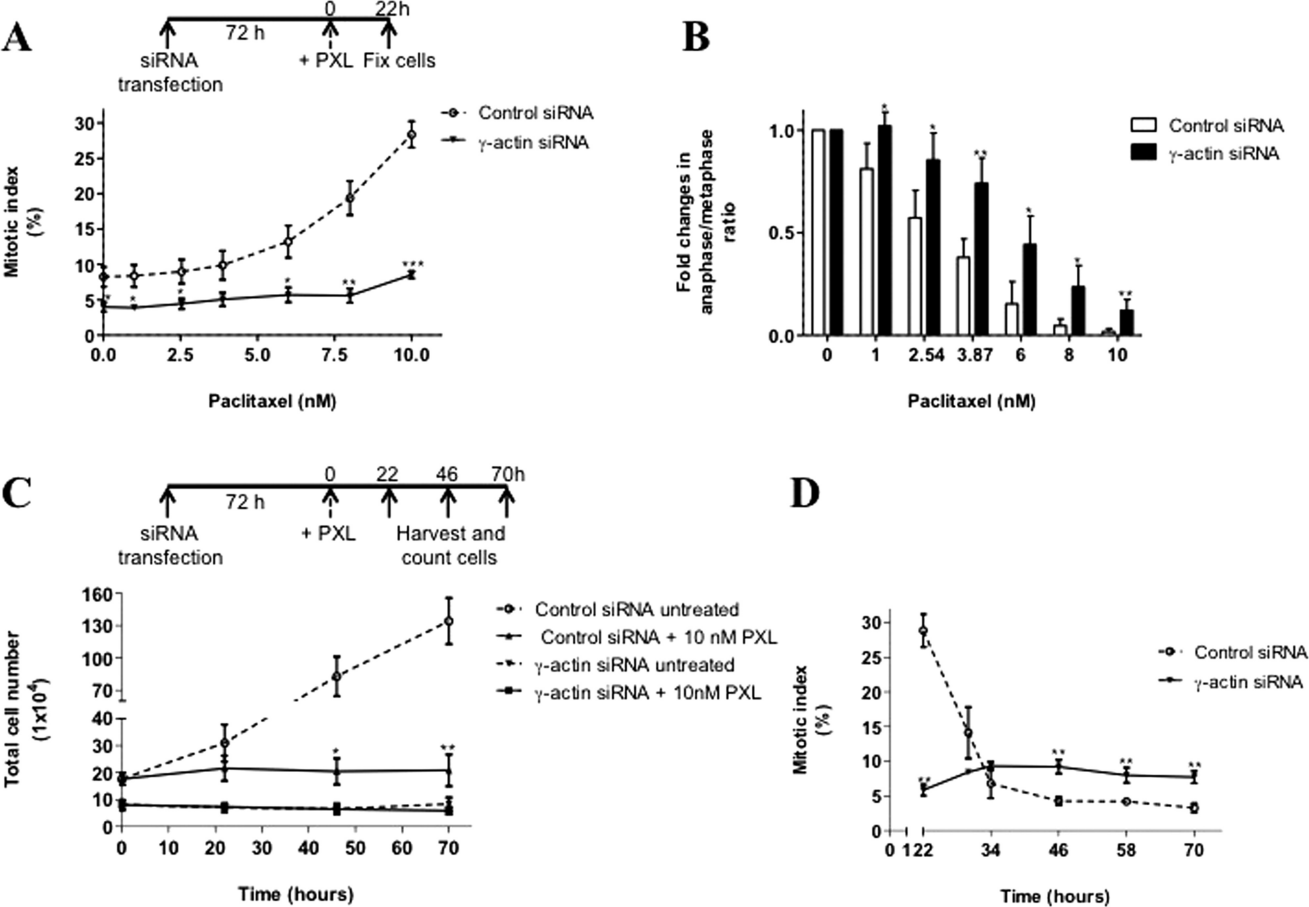
Effect of γ-actin knockdown on paclitaxel induced mitotic arrest The SH-EP GFP-βI-tubulin cells were either transfected with control siRNA or γ-actin siRNA for 72 h. The cells were incubated with increasing concentrations of paclitaxel (1–10 nM) for 22 h to attain mitotic arrest. At least 1000 interphase and mitotic cells were counted per condition. (**A**) Paclitaxel induced mitotic arrest amongst the siRNA transfected cells and the result is expressed as mitotic index. (**B**) Graph showing the dose-dependent decrease in anaphase/metaphase ratio in the control and γ-actin knockdown cells when treated with increasing concentrations of paclitaxel for 22 h. (**C**) Graph showing inhibition of cell proliferation in the control but not in the γ-actin knockdown cells following paclitaxel treatment for up to 70 h. (**D**) Mitotic index in the control and γ-actin transfectants treated with 10 nM paclitaxel for 22–70 h. Data are mean ± SEM of at least three independent experiments. *P < 0.05; ^**^P < 0.005; ^***^P < 0.001 statistically significant comparing γ-actin knockdown cells to the control.

To determine whether mitotic arrest induced by a microtubule inhibitor is impaired in the γ-actin depleted cells, the γ-actin knockdown and control cells were treated with increasing concentrations of paclitaxel for 22 h to attain mitotic arrest. A dose-dependent increase in mitotic index was observed in the control cells with the highest mitotic arrest of 28.4% observed with 10 nM paclitaxel, reflecting a 3.44-fold increase in mitotic index compared to drug-free cells (Fig. 4A). A dose-dependent increase in mitotic index was also observed in the γ-actin knockdown cells, however this occurred to a significantly lesser extent compared to the control siRNA cells (Fig. 4A). Inhibition of paclitaxel induced mitotic arrest was further validated using γ-actin siRNA duplex 1 and 2 (Supporting Information Fig. S4A). These results were supported by using FACS analysis with paclitaxel, which showed that partial depletion of γ-actin reduces the ability of paclitaxel to induce G_2_/M cell cycle arrest (data not shown). The low mitotic index could be due to the slower proliferation of the γ-actin knockdown cells, reducing paclitaxel's ability to block cells in mitosis. Paclitaxel treatment induced a dose-dependent decrease in anaphase/metaphase ratio both in the control and γ-actin knockdown cells however, the effect was more pronounced in the control siRNA cells compared to the γ-actin knockdown cells (Fig. 4B). For example, treatment with 10 nM paclitaxel reduces the anaphase/metaphase ratio in the control siRNA cells by 52.6-fold in contrast to a 8.2-fold reduction in the γ-actin knockdown cells. Similarly, less inhibition of metaphase/anaphase transition was observed in γ-actin siRNA duplex 1 and 2 cells when treated with 10 nM paclitaxel for 22 h compared to drug treated control siRNA cells (Supporting Information Fig. S4B). The increase in mitotic index and subsequent decrease in the anaphase/metaphase ratio was more pronounced in the control siRNA cells following paclitaxel treatment compared to γ-actin knockdown cells under the same conditions. This demonstrates that partial depletion of γ-actin leads to decreased sensitivity to paclitaxel. This same concentration of paclitaxel (10 nM) inhibits cell proliferation in the control siRNA cells with no significant effect in the γ-actin knockdown cells (Fig. 4C). In addition, we extended the incubation time from 22 to 70 h allowing for the γ-actin knockdown cells to complete at least one cell division in the presence of 10 nM paclitaxel. Interestingly, paclitaxel-induced mitotic arrest was considerably reduced in the γ-actin knockdown cells compared to control siRNA cells (Fig. 4D), which further supports the results observed with the 22 h paclitaxel treatment (Fig. 4A). These data clearly indicate that γ-actin is required for paclitaxel induced mitotic arrest.

We also examined the effects of this concentration of paclitaxel on microtubule organization in the γ-actin knockdown cells. Following 22 h treatment with 10 nM paclitaxel, microtubule bundling was prominent in the control siRNA cells (Supporting Information Fig. S5iii), however normal microtubule organization was still observed in the γ-actin depleted cells (Supporting Information Fig. S5iv). This concentration of paclitaxel has a dramatic effect in inducing mitotic arrest, inhibiting cell proliferation and causing microtubule bundling in the control siRNA treated cells compared to the γ-actin knockdown cells. Collectively, these data demonstrate that decreased γ-actin expression delays mitotic progression, cell proliferation and markedly reduces the ability of paclitaxel to induced mitotic arrest.

### The Microtubule Inhibitor, Paclitaxel, Further Suppressed Microtubule Dynamicity in the γ-Actin Knockdown Cells

We then investigated whether partial depletion of γ-actin would affect the ability of paclitaxel to suppress interphase microtubule dynamics. Numerous studies have shown that microtubule-targeted agents can induce mitotic arrest and inhibit cell proliferation via suppression of microtubule dynamics [Jordan et al., 1992, 1993; Yvon et al., 1999; Honore et al., 2003, 2004; Kamath and Jordan, 2003; Kamath et al., 2006; Gan et al., 2010]. The γ-actin knockdown and control siRNA cells were treated with 10 nM paclitaxel for 4 h. As shown in the preceding section, this concentration of paclitaxel (10 nM) blocked metaphase-anaphase transition in the γ-actin knockdown and the control siRNA cells (Fig. 4B) but only induced a higher mitotic arrest (Fig. 4A), and detectable microtubule bundling (Supporting Information Fig. S5) in the control siRNA cells.

Interestingly, paclitaxel treatment altered the same parameters of the microtubule dynamic instability in the control and γ-actin cells but there were differences too. Paclitaxel decreased microtubule growing rates in the control siRNA (−15.65%) and γ-actin siRNA (−22.35%) and the shortening rates in the control siRNA (−33.04%) and γ-actin siRNA (−27.52%) compared to their respective non-drug treated controls (Table II). This is consistent with decreased growth and shortening rates observed in A-498 kidney and the Caov-3 ovarian cancer cells when treated with 100 and 30 nM paclitaxel, respectively [Yvon et al., 1999] and paclitaxel treatment of H460 transfected with control and βIII-tubulin siRNAs [Gan et al., 2010]. In addition, paclitaxel treatment increased the distance-based catastrophe frequency in the control siRNA (+47.21%) and γ-actin knockdown cells (+31.57%) (Table II). Microtubule targeted drug-induced increase in distance-based frequencies have been previously reported [Honore et al., 2004; Pagano et al., 2012]. Interestingly, paclitaxel treatment of the control siRNA cells reduced microtubule time spent in growing phases (−23.57%), shortened the average growing length (−23.93%), and promoted longer pause duration (+21.57%) (Table II). In contrast, paclitaxel treatment did not significantly perturb these parameters in the γ-actin knockdown cells (Table II). Moreover, compared to the control siRNA cells, paclitaxel significantly decreased the time-based rescue frequency by 24.41% in the γ-actin knockdown cells (Table II), however time-based catastrophe frequency remained unchanged following paclitaxel treatment in both the control siRNA and γ-actin knockdown cells (Table II). Paclitaxel suppressed interphase microtubule dynamicity by a similar level in both the γ-actin knockdown (−24.43%) and control siRNA cells (−28.80%) (Table II). Although interphase microtubule dynamics was already suppressed following γ-actin knockdown, this concentration of paclitaxel (10 nM) still has the ability to further suppress interphase microtubule dynamics in the γ-actin knockdown cells. However, this concentration of paclitaxel failed to induce a greater mitotic arrest in the γ-actin knockdown cells. Importantly, paclitaxel inhibits metaphase/anaphase transition and suppressed microtubule dynamicity in both the control siRNA and γ-actin knockdown cells. These data suggest that γ-actin is required for paclitaxel induced mitotic arrest but not for paclitaxel suppression of microtubule dynamics.

**Table II:** The Effects of Paclitaxel on Parameters of Microtubule Dynamics in the γ-Actin Knockdown and Control siRNA Cells

Parameter	Control siRNA			γ-Actin siRNA		
	
	Untreated	10 nM	% Change^a^	Untreated	10 nM	% Change^a^
Mean rates (μm/min)						
Growth	8.42 ± 0.23	7.10 ± 0.44**	**−15.65**	8.46 ± 0.34	6.56 ± 0.27*	**−22.35**
Shortening	12.55 ± 0.50	8.40 ± 0.47**	**−33.04**	10.66 ± 0.29	7.72 ± 0.41***	**−27.52**
% Time spent						
Growth	38.40 ± 0.73	29.35 ± 1.01***	**−23.57**	30.64 ± 0.90	27.44 ± 3.12	NS
Shortening	22.73 ± 1.27	26.11 ± 1.98	NS	22.56 ± 0.86	25.17 ± 1.60	NS
Attenuation	38.87 ± 1.6	44.54 ± 2.98	NS	46.80 ± 1.39	47.39 ± 4.46	NS
Mean duration (min)						
Attenuation	0.17 ± 0.01	0.21 ± 0.02*	**+21.57**	0.20 ± 0.01	0.23 ± 0.02	NS
Mean length (μm)						
Growth	0.68 ± 0.03	0.52 ± 0.06*	**−23.93**	0.53 ± 0.04	0.43 ± 0.07	NS
Shortening	0.63 ± 0.05	0.55 ± 0.07	NS	0.52 ± 0.04	0.48 ± 0.06	NS
Catastrophe frequency (per min)	2.21 ± 0.17	2.18 ± 0.27	NS	2.28 ± 0.15	1.98 ± 0.16	NS
Rescue frequency (per min)	6.84 ± 0.29	5.66 ± 0.17	NS	7.26 ± 0.23	5.49 ± 0.04**	**−24.41**
Catastrophe frequency (per μm)	0.59 ± 0.04	0.87 ± 0.03**	**+47.21**	0.78 ± 0.04	1.02 ± 0.17*	**+31.57**
Rescue frequency (per μm)	0.60 ± 0.05	0.76 ± 0.04	NS	0.75 ± 0.03	0.83 ± 0.07	NS
Dynamicity (μm/min)	6.05 ± 0.18	4.31 ± 0.48**	**−28.80**	4.99 ± 0.22	3.77 ± 0.47*	**−24.43**

Values are mean ± SEM.

* *P* < 0.05,

** *P* < 0.005,

*** *P* < 0.001, statistically significant when comparing the drug treated cells to the non-drug treated cells; NS: not statistically significant. Number of microtubules measured was from 120 to 360 from 30 to 90 cells.

a Percentage change was calculated relative to the non-drug treated cells.

## Discussion

Previous studies have identified γ-actin alterations in association with resistance to microtubule targeting agents [Verrills et al., 2003, 2006a, b]. This article sought to address whether γ-actin affects microtubule function and the action of microtubule-targeted agents. Herein, we demonstrate for the first time that partial depletion of γ-actin suppresses interphase microtubule dynamics, and delays metaphase–anaphase transition. These findings link γ-actin and microtubule dynamics and potentially influence the action of microtubule-targeted agents.

It is unclear whether suppression of microtubule dynamics due to γ-actin knockdown is via a direct effect of decreased γ-actin expression on microtubule dynamics or via other regulatory proteins that regulate microtubule polymerization and depolymerization. Association between drug-induced suppression of interphase microtubule dynamics and increased microtubule stability and acetylation has been previously demonstrated [Mohan and Panda, 2008]. However, suppression of microtubule dynamics observed in the γ-actin knockdown cells is not associated with an increase in microtubule stability, since microtubules in the γ-actin knockdown cells were less acetylated and detryrosinated compared to microtubules in the control siRNA cells. In contrast to our study, a correlation between microtubule stability and increased microtubule detryrosination has been observed in other cell types [Gundersen et al., 1987; Webster et al., 1987]. It is not clear whether suppression of γ-actin expression inhibits tubulin detyrosination or causes disassembly of detyrosinated microtubules. Of interest, tubulin detyrosination has been shown to inhibit the activity of microtubule depolymerizing kinesins, mitotic centromere-associated kinesin (MCAK) and KIF2A which resulted in stabilization of microtubules [Peris et al., 2009]. We have not excluded the possibility that MCAK or KIF2A expression is increased in the γ-actin knockdown cells, which in turn could inhibit tubulin detyrosination. The significance of less detyrosination and acetylation of microtubules due to suppression of γ-actin expression is not clearly understood and requires further investigation.

The fact that distance-based frequencies were altered but not time-based frequencies (with constant growth rate) in the γ-actin knockdown cells indicate it is due to an increase in microtubule pauses. In addition, reduced microtubule shortening rates and time spent in microtubule growth following γ-actin knockdown reflects the increase in microtubule pause/attenuation. Microtubule ends pause and tether to the actin cortex which is thought to be aided by several proteins, such as the +TIPS and microtubule-actin cross-linker proteins. Microtubule plus-end tracking proteins, such as EB1 and CLIP170 bind to the distal ends of the microtubule, stabilizing the microtubules and promoting microtubule growth, and preventing catastrophe [Carvalho et al., 2003; Howard and Hyman, 2003; van der Vaart et al., 2009]. Once the microtubule ends reached the cell periphery, +TIPS dissociate from the microtubule distal tips allowing depolymerization of microtubules. As shown in Fig. 2, the microtubule ends in the γ-actin knockdown cells are less dynamic and paused at the cell periphery for a prolonged period indicating that the microtubule ends are highly captured at the cell cortex. Since γ-actin is localized to the cell periphery of SH-EP cells and is depleted from the cell periphery following γ-actin siRNA treatment [Shum et al., 2011]. It is possible that loss of γ-actin from the cell periphery in the γ-actin knockdown cells perturbs the interaction between +TIPS such as CLIP-associating protein (CLASP) and the microtubule ends at the cell cortex. Therefore, contributing to microtubule prolonged capturing at the cell cortex resulting in enhanced pause/attenuation. CLASP1 and CLASP2 have been shown to accumulate at the microtubule ends near the cell periphery and play a role in regulating microtubule stability [Mimori-Kiyosue et al., 2005].

Furthermore knockdown of γ-actin may have altered the function of microtubule-actin cross-linker proteins, such as actin crosslinking family 7, which have been shown to regulate microtubule dynamics and microtubules tethering to the cortical actin sites [Kodama et al., 2003]. Loss of γ-actin from the cell periphery together with loss of lamella and lamellipodial-like structures [Shum et al., 2011], may have restricted the microtubules from tethering to the cortical actin sites which is consistent with a reduction in the average microtubule growing length observed in the γ-actin knockdown cells. However, we cannot exclude the possibility that the decrease in microtubule growing length could be a consequence of prolonged microtubule pausing at the cell cortex. Decreases in the period of microtubule growth in γ-actin knockdown cells may lead to EB1 or other +TIPS being retained by the microtubule tips at the cell cortex which in turn results in enhanced microtubule pause/attenuation. Further studies are required to determine the effect of γ-actin knockdown on function of +TIPs. These changes in microtubule dynamic parameters due to depletion of γ-actin may have a combination of effects: fewer microtubules are able to reach the cell cortex; and those microtubules that manage to reach the cell cortex are being captured for a prolonged period.

The observation that distance-based frequencies were increased in the γ-actin knockdown cells appears to correlate with our recently reported reduced cell migration of SH-EP cells [Shum et al., 2011]. An increase in distance-based catastrophe frequency and reduction in EB1 accumulation at the microtubule plus ends has been linked to the anti-migratory effect of the microtubule-targeted agent, Epothilone B in glioblastoma cells [Pagano et al., 2012]. The growing microtubules are captured and stabilized at the cell cortex during cell migration and an increase in catastrophe frequency would reduce the number of microtubules reaching the leading edge hence impair cell migration [Pagano et al., 2012].

Our finding that there is no correlation between suppression of microtubule dynamics and increased mitotic arrest in the absence or presence of paclitaxel in the γ-actin knockdown cells, suggest that suppression of microtubule dynamics may not always lead to mitotic arrest. This is in contrast with previous published data demonstrating that suppression of interphase microtubule dynamics induced by microtubule-targeted agents leads to mitotic arrest [Honore et al., 2003, 2004; Kamath and Jordan, 2003; Kamath et al., 2006; Gan et al., 2010]. It is possible that the low levels of paclitaxel-induced mitotic arrest in the γ-actin knockdown cells are in part due to reduce cell proliferation decreasing the effectiveness of paclitaxel in blocking cells in mitosis. However, prolonged incubation with paclitaxel still fails to significantly increase the number of γ-actin knockdown cells arrested in mitosis, even though the γ-actin knockdown cells have undergone at least one complete cell division in the presence of paclitaxel. Therefore, inhibition of paclitaxel-induced mitotic arrest is also due to the effect of γ-actin depletion rather than just a consequence of reduced cell proliferation. A limitation with our study is that we are measuring interphase microtubule dynamics and the effects of partial suppression of γ-actin on kinetochore microtubule dynamics are unknown.

Interestingly, the inhibition of paclitaxel induced mitotic arrest observed in the γ-actin knockdown cells is consistent with the resistance phenotype that we previously described in the SH-EP γ-actin knockdown cells against paclitaxel [Verrills et al., 2006b]. It has been suggested that anti-microtubule agents' induce mitotic arrest by increasing microtubule bundling and polymer levels [Jordan et al., 1992, 1993]. Microtubule bundling was evident in the control cells treated with paclitaxel but not in the γ-actin knockdown cells. This correlates with our previous finding that suppression of γ-actin partially inhibits paclitaxel induced tubulin polymerization [Verrills et al., 2006b]. Paclitaxel induced mitotic arrest in the control siRNA cells is probably a consequence of (1) suppression of microtubule dynamics and (2) increased microtubule bundling.

In addition to mitotic arrest, previous studies have shown that microtubule targeted agents induce suppression of microtubule dynamics with a concomitant block in metaphase–anaphase transition [Jordan et al., 1992, 1993; Dhamodharan et al., 1995; Yvon et al., 1999; Honore et al., 2003, 2004; Kamath and Jordan, 2003; Kamath et al., 2006]. Does suppression of interphase microtubule dynamics lead to inhibition of metaphase–anaphase transition? We showed that either knockdown of γ-actin or paclitaxel treatment of γ-actin knockdown cells suppressed microtubule dynamics with no major increase in mitotic arrest and the only common observation was the inhibition of metaphase-anaphase transition. It is possible that inhibition of metaphase–anaphase transition is a consequence of suppression of interphase microtubule dynamics. Importantly, paclitaxel suppression of microtubule dynamics and inhibition of metaphase–anaphase transition may not require γ-actin, however γ-actin is required for paclitaxel induced mitotic arrest.

Furthermore, same parameters of the microtubule dynamics were affected by γ-actin knockdown and paclitaxel treatment of the γ-actin knockdown cells such as a decrease in microtubule shortening rates and increase in length-based catastrophe frequency. Interestingly, paclitaxel treatment did not significantly affect the pause duration and period of microtubule growth in the γ-actin depleted cells compared to the effect of paclitaxel in the control siRNA cells suggesting that knockdown of γ-actin already induced maximal changes in these parameters.

This is the first demonstration that alterations in γ-actin regulate interphase microtubule dynamics, mitotic progression and the ability of paclitaxel to induce mitotic arrest. This study identified that γ-actin is required for proper microtubule function and paclitaxel induced mitotic arrest. Further studies are required to identify how γ-actin modulates interphase microtubule dynamics as this study demonstrated the first link between γ-actin and microtubule dynamics.

## Materials and Methods

### Cell Culture and Plasmid Transfection

The neuroblastoma SH-EP cells were stably transfected with GFP-βI-tubulin (encoded by the TUBB gene, accession NM_178014) plasmid to visualize the microtubules when performing time-lapse fluorescence microscopy. We previously used this plasmid and approach to examine microtubule dynamics in lung cancer cells [Gan et al., 2010]. Briefly, the SH-EP cells were resuspended in electroporation buffer (20 mM HEPES pH 7.05; 137 mM NaCl; 5 mM KCl; 0.7 mM Na_2_HPO_4_; and 6 mM Glucose) at 3 × 10^6^ cells/mL and 30 μg of the DNA plasmid was added to the mix and pulse at 360V/500 μF. Following 48 h post-transfection, stable transfectants were selected in media containing 1 mg/mL G418 (Geneticin, Life Technologies, Mulgrave, Victoria, Australia). Individual colonies were screened for GFP expression by fluorescence microscopy and then sorted by FACS to select for high expressing GFP-βI-tubulin cells.

The SH-EP GFP-βI-tubulin cells were maintained as a monolayer in Dulbecco's Modified Eagle Medium (Life Technologies) supplemented with 10% fetal calf serum (FCS) and grown in a humidified incubator at 37°C and 5% CO_2_. Cell lines were routinely screened and found to be free of mycoplasma.

### Cytotoxic Drugs

Paclitaxel (Merck Millipore, Darmstadt, Germany) was prepared at a stock concentration of either 300 μM or 2 mM in DMSO.

### γ-Actin gene knockdown

Transfection of γ-actin siRNA was carried out as previously described [Verrills et al., 2006b]. Briefly, the SH-EP GFP-βI-tubulin cells were plated onto either six well plates or glass coverslips at 6 × 10^4^ cells per well and transiently transfected with either the All Stars negative control siRNA (100 nM, Qiagen, Valencia, CA) or γ-actin siRNA (encoded by the *ACTG1* gene, accession NM_001614) (5′-AAGAGATCGCCGCGCTGGTCA-3′, 100 nM, Qiagen) [Harborth et al., 2001] using Lipofectamine 2000 (Life Technologies), according to manufacturer's instructions. In order to confirm the uptake of siRNA into the cells when performing time-lapse fluorescence microscopy, the γ-actin and All Stars negative control siRNAs were labeled at the 3′-end with Rhodamine (Qiagen).

### Immunofluorescence Microscopy

To assess microtubule structure, SH-EP GFP-βI-tubulin cells were either transfected with control siRNA or γ-actin siRNA for 72 h as described above. The siRNA transfected cells were fixed with ice-cold methanol for 10 min and then stained with monoclonal antibodies against anti-tubulin acetylated (clone 6-11B-1) (Sigma-Aldrich, St Louis, MO) and polyclonal anti-tubulin detyrosinated (Glu-tubulin, Merck Millipore). The cells were stained with secondary antibody Alexa-Fluor 555 anti-mouse (Life Technologies) or Alexa-Fluor 568 anti-rabbit (Life Technologies) then counterstained with 4,6 diamidino-phenylindole (DAPI, Vector Laboratories, Burlingame, CA). Images were acquired using an Olympus Fluoview FV1000 confocal microscope (Olympus, Tokyo, Japan) equipped with an oil immersion plan apochromat 1.4 NA 100× objective lens. A *z* series of images of 0.4 μm per section were collected using sequential scanning. Images were processed using the Olympus Fluoview software (Olympus).

### Western Blotting

Twenty micrograms of protein lysates obtained from the siRNA transfected cells were separated on 4–15% SDS–PAGE gel (Bio-Rad, Hercules, CA), electrotransfer to nitrocellulose membranes. The membranes were probed with monoclonal antibodies against acetylated tubulin (clone 6-11B-1) and tyrosine tubulin, (Tyr-tubulin, clone TUB1A2) (Sigma-Aldrich), polyclonal antibodies against detyrosinated tubulin and GAPDH (clone 6C5, Abcam, Cambridge, UK) as a control for equal loading. Proteins were detected by ECL Plus (GE Healthcare, Uppsala, Sweden) and membranes were scanned using the Typhoon (GE Healthcare).

### Time-Lapse Microscopy and Image Acquisition

SH-EP GFP-βI-tubulin cells were plated at 6 × 10^4^ cells on glass coverslips and transfected with siRNA for 72 h as described above. The cells were incubated with or without paclitaxel for 4 h to attain equilibrium in the intracellular drug concentration. Preparation of cells for time-lapse microscopy and image acquisition were performed as previously described with minor modifications [Pagano et al., 2012]. Briefly, cells grown on coverslips were assembled onto a double coverslip chamber containing recording media (RPMI 1640 culture media without phenol red or sodium bicarbonate and supplemented with 4.5 g/L glucose, 25 mM HEPES, 10% FCS, and 0.1 mg/mL Vitamin C to prevent photo bleaching) and maintained at 37 ± 1°C. The cells were observed using a Leica DM-IRBE fluorescence microscope fitted with a 100× objective lens. Only cells that had taken up the Rhodamine labeled siRNA were acquired for microtubule dynamic analysis. Thirty-one images were acquired per cell at 4 s intervals using the CCD camera CoolsnapFX (Princeton Instruments, Trenton, NJ) digital camera driven by the Metamorph software (Molecular Devices, Downingtown, PA) and a maximum of 1 h was spent acquiring images from each coverslip.

### Analysis of Microtubule Dynamic Instability

Analysis of microtubule dynamic instability was carried out as previously described [Kamath and Jordan, 2003; Honore et al., 2004; Gan et al., 2010].The plus ends of individual microtubules in interphase cells were tracked over time using the Metamorph software and results graphed as a “life history plot,” displaying microtubule length over time. Criteria for determining the parameters of microtubule dynamics have been previously described [Honore et al., 2004]. Linear regression was used to calculate the growth and shortening rates and changes in length of ≥0.5 μm were considered as a growth or shortening event, whereas changes in length of <0.5 μm were considered as a attenuation or pause event. The time-based catastrophe frequency (transitions from growth or pause to shortening) was calculated by dividing the number of catastrophes per microtubule by the time spent in growth and pause. The length-based catastrophe frequency was calculated by dividing the number of catastrophes by the total length grown for each microtubule. The time-based rescue frequency (transitions from shortening to growth or pause) was calculated by dividing the number of rescues per microtubule by the total time spent in shortening. The length-based frequency was calculated by dividing the total number of rescues by the total length of shortening for each microtubule. Dynamicity is the total length grown and shortened divided by life span of the microtubules. Forty microtubules were analyzed per each condition from at least three independent experiments. Results are mean and SEM of at least three independent experiments.

### Mitotic Progression

SH-EP GFP-βI-tubulin cells were plated onto six well plates and transfected with siRNA as described above. Assessing of mitotic progression was carried out as previously described with modifications [Po'uha et al., 2010]. Seventy-two hours post-siRNA transfection, the cells were either fixed with formaldehyde as described below or treated with increasing concentrations of paclitaxel (1–10 nM, Merck Millipore) for 22 h or with 10 nM paclitaxel for various time points (22–70 h) to reach mitotic arrest. The cells were fixed with 3.7% formaldehyde in PBS, which was added directly to the medium for 30 min, followed by PBS washes and the cells were stained with DAPI (Vector Laboratories) to visualize the chromosomes. Cells were observed using an Axiovert 200 M fluorescent microscope (Zeiss, Oberkochen, Germany) coupled to an AxioCamMR3 camera and driven by the Axio Vision software (Zeiss) fitted with a 40× objective lens. At least 1000 interphase and mitotic cells were counted per condition from at least three independent experiments. Mitotic index was calculated by dividing the total number of mitotic cells by the total number of interphase and mitotic cells counted.

### Determining Cell Proliferation

The SH-EP GFP-βI-tubulin cells were transfected with either control or γ-actin siRNA and harvested every 24 h and total cells were counted by trypan blue exclusion to determine growth rate. Doubling time for the control and γ-actin transfected cells was determined from their individual growth curve. To determine whether paclitaxel further inhibits cell proliferation in the γ-actin knockdown cells, the siRNA transfected cells were treated with 10 nM paclitaxel for up to 70 h and harvested at 22, 46 and 70 h and total cells were counted by trypan blue exclusion.

### Statistical Analysis

Statistical analysis was performed using two sided, unpaired Student's *t*-test and values expressed as mean ± SEM of at least three independent experiments. *P* < 0.05 was considered statistically significant.

## Supplementary material

Supporting Information Figure 1. The SH-EP GFP-β1-tubulin cells were transfected with either the control siRNA or α-actin siRNA duplex 1 and 2 for 72 hours and cells were harvested for western blot analysis or immunostained for confocal image analysis. A. Western blot analysis showing knockdown of α-actin in SH-EP GFP-βI-tubulin cells by α-actin siRNA duplex 1 and 2. Graph showing β-actin relative expression. Data is mean ± SEM of three independent experiments. *P<0.05, statistically significant comparing β-actin knockdown cells to the control siRNA cells.

Supporting Information Figure 2. SH-EP GFP-βI-tubulin cells were transfected with either the control siRNA or α-actin siRNA duplex 1 and 2 for 72 hours and cells were assessed using confocal microscopy and western blotting. BA. Representative images of detryrosinated and acetylated microtubules in the siRNA transfected cells. Images were taken using a laser scanning confocal microscope. Scale bar, 10 μm. CB. Western blotting analysis of detyrosinated, tyrosinated and acetylated tubulin levels in the siRNA transfected cells. Graph shows the relative protein expression. Data is mean ± SEM of three independent experiments. **P<0.005 statistically significant comparing α-actin knockdown cells to the control siRNA cells.

Supporting Information Figure 3. A. Western blot analysis showing partial suppression of γ-actin in the SH-EP GFP-βI-tubulin cells using the rhodamine labeled γ-actin siRNA. Data is mean ± SEM of three independent experiments. *P<0.05 statistically significant comparing γ-actin knockdown cells to the control siRNA cells. B. Representative images of the SH-EP GFP-βI-tubulin cells transfected with Rhodamine labeled siRNAs (red) for 24 hours showing that the SH-EP GFP-βI-tubulin cells have a very good transfection efficiency (∼100%). Scale bar, 100 μm

Supporting Information Figure 4. Inhibition of paclitaxel induced mitotic arrest (A) and less blockage of metaphase/anaphase transition (B) in the γ-actin siRNA duplex 1 and 2 cells treated with 10 nM paclitaxel for 22 hours. A total of 1000 interphaahse and mitotic cells were counted per condition. Data is mean ± SEM of four independent experiments. **P<0.005, ***P<0.001 statistically significant comparing γ-actin knockdown cells to the control siRNA cells.

Supporting Information Figure 5. Effect of α-actin knockdown in paclitaxel induced microtubule bundling. The siRNA transfected cells were untreated or treated with 10 nM paclitaxel for 22 hours, followed by indirect immunofluorescence staining with a-tubulin antibody to visualize the microtubule network. Scale bar, 10 μm.
