# Supplementary figures and images for: Partial Depletion of Gamma-Actin Suppresses Microtubule Dynamics
Source: Cytoskeleton (Hoboken). 2013 Jan 17;70(3):148–60. doi: 10.1002/cm.21096 (PMC3613743; doi:10.1002/cm.21096)

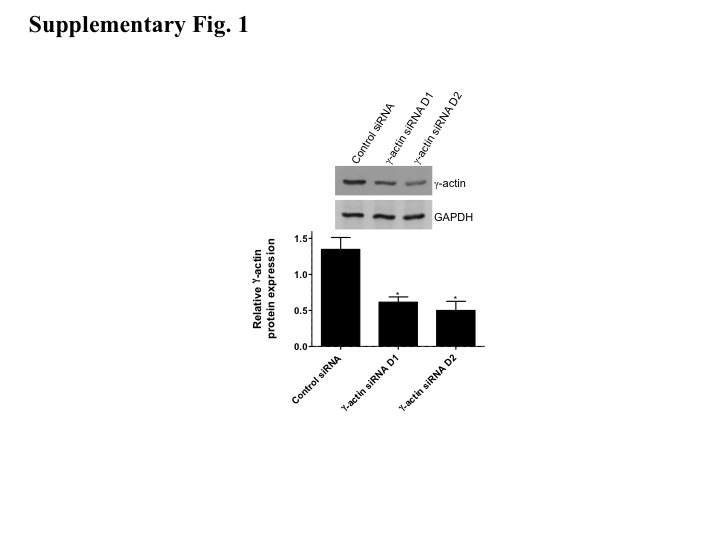

Supplement: Supplementary file 1 [file cm0070-0148-SD1.jpg]

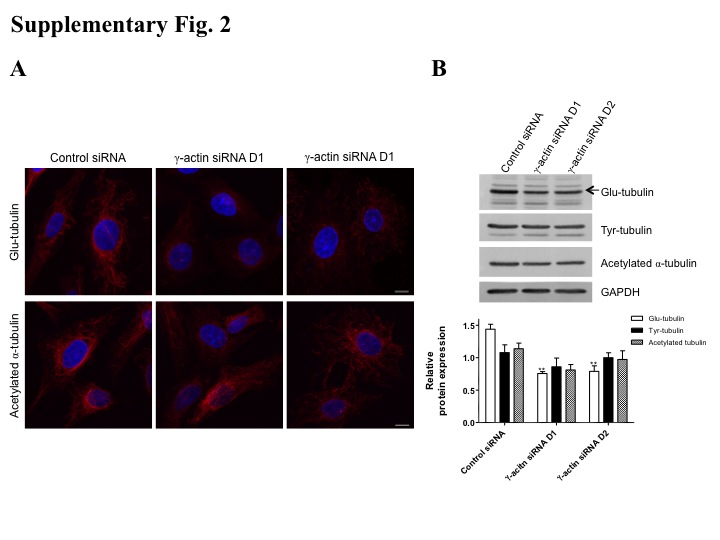

Supplement: Supplementary file 2 [file cm0070-0148-SD2.jpg]

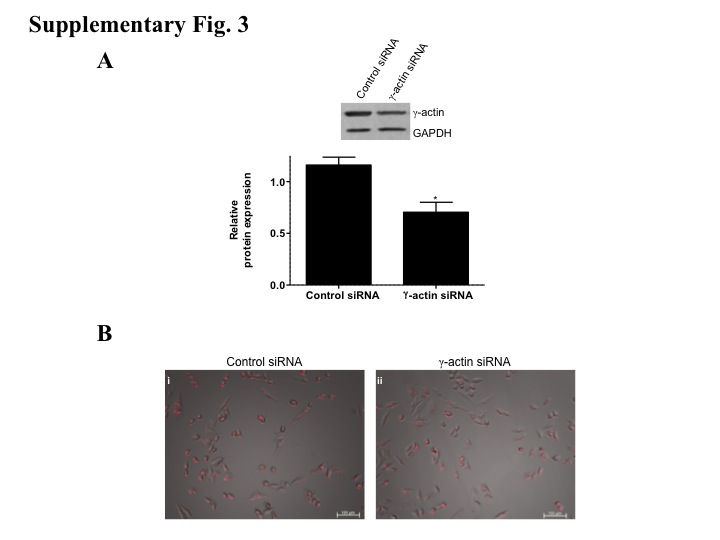

Supplement: Supplementary file 3 [file cm0070-0148-SD3.jpg]

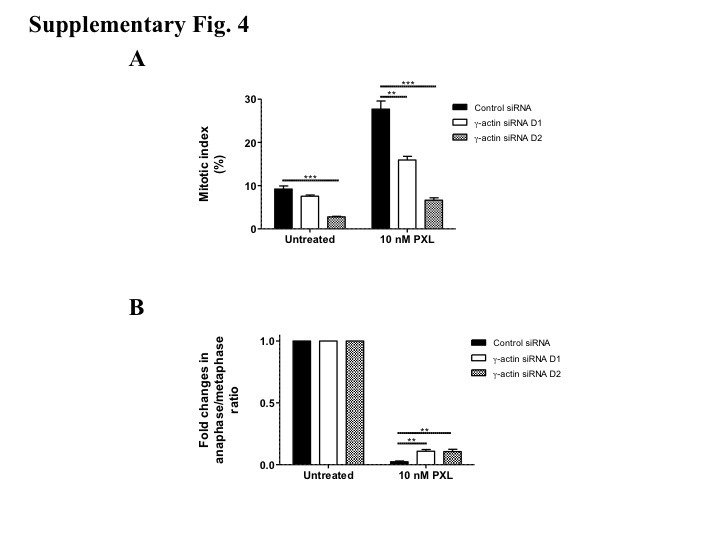

Supplement: Supplementary file 4 [file cm0070-0148-SD4.jpg]

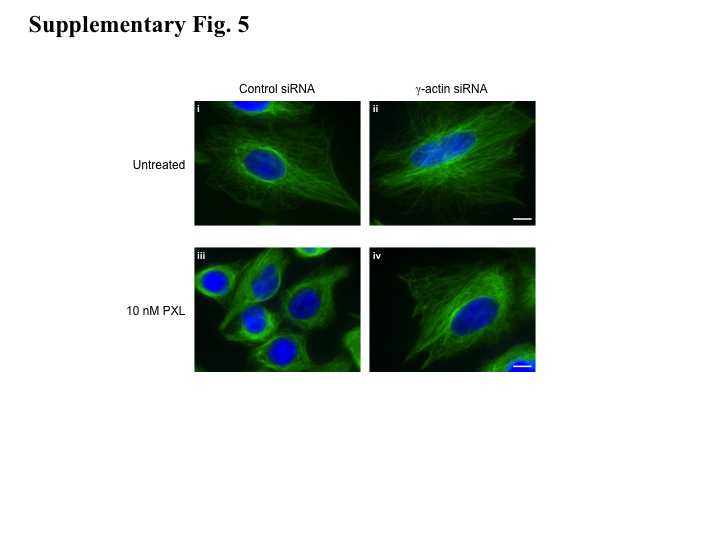

Supplement: Supplementary file 5 [file cm0070-0148-SD5.jpg]
