# Supplementary material for: Partial Depletion of Gamma-Actin Suppresses Microtubule Dynamics
Source: Cytoskeleton (Hoboken). 2013 Jan 17;70(3):148–60. doi: 10.1002/cm.21096 (PMC3613743; doi:10.1002/cm.21096)
Supplement: Supplementary file 6 [file cm0070-0148-SD6.doc]

**Supplementary Methods**

**Western Blotting**

SH‑EP GFP‑ βI‑tubulin cells were either transfected with rhodamine labeled control siRNA or γ-actin siRNA for 72 hours as described above. Ten micrograms of protein lysates were separated on 12% SDS-PAGE gel, electrotransfer to nitrocellulose membranes. The membranes were probed with polyclonal antibody against γ-actin [Schevzov et al. 2005] and monoclonal antibody against GAPDH (clone 6C5, Abcam) as a control for equal loading. Proteins were detected by ECL Plus and membranes were scanned using the Typhoon (GE Healthcare).

**Immunofluorescene microscopy**

SH‑EP cells were plated onto 4 well glass chambered slides and transfected with siRNA as described above. Seventy‑two hours post‑siRNA transfection, the cells were treated with 10 nM paclitaxel (Calbiochem, Merck Biosciences). The cells were then fixed with 100% ice-cold methanol followed by staining with monoclonal anti-α-tubulin (Sigma-Aldrich) then with anti-mouse-Alexa Fluor 488 secondary antibody (Molecular Probes-Invitrogen) to visualize the microtubule network and counterstained with 4,6 diamidino‑phenylindole (DAPI) to visualize the chromosomes. Images were acquired using an Axiovert 200 M fluorescent microscope (Zeiss, Oberkochen, Germany) coupled to an AxioCamMR3 camera and driven by the Axio Vision software (Zeiss) fitted with a plan apochromat 1.4 N.A. X100 objective lens.

γ‑Actin gene knockdown

Additional γ‑actin siRNAs were purchased from Dharmacon (Lafayette, CO), γ‑actin siRNA duplex 1 (5′- GAGAAGAUGACUCAGAUU-3′, 25 nM, Dharmacon) [Baranwal et al. 2012] and γ‑actin siRNA duplex 2 (5′-GAGCCGUGUUUCCUUCCAU-3′, 25 nM) [Baranwal et al. 2012].
